# Respiratory tract infections and risk factors for infection in a cohort of 330 patients with axial spondyloarthritis or psoriatic arthritis

**DOI:** 10.3389/fimmu.2022.1040725

**Published:** 2022-10-26

**Authors:** Natalie Frede, Eva Rieger, Raquel Lorenzetti, Alexandra Nieters, Ana C. Venhoff, Carolin Hentze, Marcus von Deimling, Nora Bartholomä, Jens Thiel, Reinhard E. Voll, Nils Venhoff

**Affiliations:** ^1^ Department of Rheumatology and Clinical Immunology, Medical Center - University of Freiburg, Faculty of Medicine, University of Freiburg, Freiburg, Germany; ^2^ Institute for Immunodeficiency, Center for Chronic Immunodeficiency, Medical Center - University of Freiburg, Faculty of Medicine, University of Freiburg, Freiburg, Germany; ^3^ Division of Rheumatology and Clinical Immunology, Medical University Graz, Graz, Austria

**Keywords:** respiratory tract infections, infection risk, antibiotic therapy, axial spondyloarthritis (AxSpA), psoriatic arthritis (PsA), spondyloarthritis (SpA)

## Abstract

Respiratory tract infections (RTIs) are the most common infections in patients with rheumatic diseases under immunosuppressive treatment and may contribute to morbidity and mortality as well as increased healthcare costs. However, to date only limited data on infection risk in spondyloarthritis (SpA) patients are available. In this study we assessed the occurrence of respiratory tract infections in a monocentric real-world cohort consisting of 330 patients (168 psoriatic arthritis and 162 axial spondyloarthritis patients) and determined factors associated with increased infection risk. Out of 330 SpA patients, 89.3% had suffered from ≥ 1 upper respiratory tract infection (URTI) and 31.1% from ≥ 1 lower respiratory tract infection (LRTI) within the last two years. The most common URTIs were rhinitis and laryngitis/pharyngitis with 87.3% and 36.1%, respectively. Bronchitis constituted the most common LRTI, reported in 29.7% of patients. In a multivariate binomial logistic regression model occurrence of LRTI was associated with chronic lung disease (OR 17.44, p=0.006), glucocorticoid therapy (OR 9.24, p=0.012), previous history of severe airway infections (OR 6.82, p=0.013), and number of previous biological therapies (OR 1.72, p=0.017), whereas HLA B27 positivity was negatively associated (OR 0.29, p=0.025). Female patients reported significantly more LRTIs than male patients (p=0.006) and had a higher rate of antibiotic therapy (p=0.009). There were no significant differences between axSpA and PsA patients regarding infection frequency or antibiotic use. 45.4% of patients had required antibiotics for respiratory tract infections. Antibiotic therapy was associated with smoking (OR 3.40, p=0.008), biological therapy (OR 3.38, p=0.004), sleep quality (OR 1.13, p<0.001) and age (OR 0.96, p=0.030). Hypogammaglobulinemia (IgG<7g/l) was rare (3.4%) in this SpA cohort despite continuous immunomodulatory treatment.

Awareness of these risk factors will assist physicians to identify patients with an increased infection risk, who will benefit from additional preventive measures, such as vaccination and smoking cessation or adjustment of DMARD therapy.

## Introduction

Respiratory tract infections (RTIs) constitute the most common infections in patients with rheumatic diseases, especially under immunosuppressive treatment (1, 2). RTIs may cause significant morbidity with reduced quality of life (QOL), increased economic loss and healthcare costs and may lead to interruption of DMARD therapy (3, 4). Additionally, serious infections may contribute to mortality in at-risk patients (5).

However, studies on infection risk in rheumatic diseases have mainly focussed on patients with rheumatoid arthritis (RA). In RA, especially a previous history of severe infections, age and glucocorticoid treatment have been identified as risk factors for infections (6). Further risk factors include number of previous DMARD therapies, comorbidities and frailty or impaired functional capacity (7). However, it has also been postulated that RA itself might confer an increased infection risk (8, 9). Thus, it is unclear whether these data are directly transferable to other rheumatic diseases.

Additionally, epidemiological and host factors such as smoking, obesity, low vitamin D serum level, as well as comorbidities such as chronic lung disease, chronic kidney disease or diabetes mellitus may contribute to an increased infection risk (3, 10–14).

Another important component mediating frequency of infections is the humoral immune system, which plays a major role in protection against respiratory infections. It is well-established that continued immunosuppressive therapy may contribute to secondary antibody deficiency with immunoglobulin serum levels decreasing over time under certain immunosuppressants (15).

To date, only limited data on infection risk in spondyloarthritis (SpA) patients are available. Biologics have revolutionized the treatment of spondyloarthritides. However, biological therapy has a major impact on immune function, and infections constitute important side effects. Data are mainly available for anti-TNF therapy, where the infection risk seems to decline over time (16). Data on infection risk are mostly derived from clinical trials with highly preselected patient cohorts, whereas real-world data are scarce. Wallis et al. demonstrated that the lung was the most common infection site in axial spondyloarthritis patients under TNF treatment (17).

In this study, we assessed infection frequency in a monocentric patient cohort of 330 spondyloarthritis patients consisting of 168 PsA and 162 SpA patients with a special focus on respiratory tract infections and determined factors associated with occurrence of respiratory infections as well as preventive measures. Furthermore, we assessed differences between axSpA and PsA as well as female and male patients.

## Materials and methods

### Patient cohort and data collection

Questionnaire-based screening and retrospective medical chart analysis was performed in a monocentric cohort of 330 patients with SpA comprising 168 psoriatic arthritis (PsA) and 162 axial spondyloarthritis (axSpA) patients recruited from the Rheumatology outpatient clinic of University Medical Center Freiburg, Freiburg, Germany between 04/2020 and 03/2022. Only patients with a confirmed diagnosis of either PsA or axSpA were included, PsA patients had to fulfil the Classification of Psoriatic Arthritis (CASPAR) criteria, axSpA patients the modified New York (mNY) or Assessment of Spondyloarthritis International Society (ASAS) criteria. Patients were consented according to local ethics guidelines. This study was conducted under the ethics protocols 190/17 and 37/17 (ethics committee of the University of Freiburg, Germany). A detailed description of the patient cohort can be found in the results section.

To assess for infections, the AWIS RTI score questionnaire was used, which constitutes a self-administered questionnaire developed by the Center for Chronic Immunodeficiency, Freiburg, Germany (18). With the help of this questionnaire, frequency and duration of individual URTIs and LRTIs (i.e. sinusitis, rhinitis, pharyngitis/laryngitis, otitis media, tonsillitis, flu-like infection, bronchitis, pneumonia, pleuritis) within the last two years as well as antibiotic treatment for these RTIs were assessed. Furthermore, the questionnaire includes items on the previous history of severe infections in general, infection-related hospitalisations, severe RTIs, subjective susceptibility to RTIs as well as other indicators of susceptibility to RTIs, comorbidities and history of lung and otolaryngeal operations. A translated version of the full questionnaire can be found in the supplemental material of the original publication. Non-response at item-level was between 5.7% and 9.1%. To assess functional capacity FFbH (Funktionsfragebogen Hannover) questionnaire was used and sleep quality was assessed by RIS questionnaire (Regensburg Insomnia Scale) (19, 20).

Questionnaire data were combined with clinical data from retrospective medical chart analysis, where correct diagnosis was confirmed and information on demographic data such as age, body weight, height, body mass index (BMI), comorbidities, as well as previous and current medication were collected. Regarding comorbidities, data on chronic lung disease, chronic kidney disease, diabetes mellitus, as well as previous or current malignant disease were collected to assess or rule out potential confounding. Chronic lung diseases occurring in this cohort included asthma, chronic obstructive pulmonary disease/chronic bronchitis, one case of non-specific interstitial pneumonia (NSIP) and one case of bronchiectasis. Frequencies of comorbidities are given in **Table 1**. Regarding biological therapies, only classes with ≥ 3 substances licensed in the indications of axSpA/PsA/psoriasis were considered for subgroup analysis, i.e. TNF inhibitors and IL17 inhibitors. Laboratory CRP and, if available, immunoglobulin serum concentrations were collected from the last outpatient clinic visit.

**Table 1:** Clinical description of patient cohort and frequency of infections.

	Total (n=330)	axSpA (n=162)	PsA (n=168)	p-value
Age, years, mean (SD)	53.3 (14.0)	49.0 (14.3)	57.4 (12.4)	<0.001
Male, n (%)/Female, n (%)	183 (55.5%)/ 147 (44.5%)	94 (58.0%)/ 68 (42.0%)	89 (53.0%)/ 79 (47.0%)	0.356
BMI, kg/m^2^, mean (SD) (n=308)	27.3 (6.15)	27.1 (6.92)	27.4 (5.43)	0.652
Smokers, n (%) (n=311)	69 (22.2%)	42 (29.0%)	27 (16.3%)	0.007
HLA B27, n (%) (n=244)	128 (52.5%)	105 (70.5%)	23 (24.2%)	<0.001
Disease duration, years, mean (SD) (n=324)	11.3 (9.29)	11.8 (10.7)	10.8 (7.62)	0.343
CRP, mg/l, mean (SD) (n=328)	6.10 (11.7)	6.85 (15.8)	5.38 (5.19)	0.255
Disease domains:				
Axial involvement, n (%) (n=329)	220 (66.9%)	162 (100%)	58 (34.7%)	<0.001
Peripheral arthritis, n (%) (n=328)	273 (83.2%)	111 (69.4%)	162 (96.4%)	<0.001
Enthesitis, n (%) (n=329)	134 (40.7%)	61 (37.9%)	73 (43.5%)	0.305
Daktylitis, n (%) (n=330)	84 (25.5%)	10 (6.2%)	74 (44.0%)	<0.001
Therapy: (n=330)				
csDMARD, n (%)	114 (34.5%)	30 (18.5%)	84 (50.0%)	<0.001
bDMARD, n (%)	224 (68.2%)	123 (75.9%)	102 (60.7%)	0.003
Anti-TNF, n (%)	145 (64.4%)	91 (74.0%)	54 (52.9%)	<0.001
Anti-IL17, n (%)	67 (29.8%)	30 (24.4%)	37 (36.3%)	0.509
Glucocorticoids, n (%)	29 (8.8%)	14 (8.6%)	15 (8.9%)	0.983
NSAID monotherapy, n (%)	35 (10.6%)	25 (15.4%)	10 (6.0%)	0.983
Vitamin D substitution, n (%)	170 (51.5%)	72 (44.4%)	98 (58.3%)	0.012
Comorbidities: (n=330)				
Chronic lung disease, n (%)	41 (12.4%)	13 (8.0%)	28 (16.7%)	0.017
Chronic kidney disease, n (%)	31 (9.5%)	12 (7.4%)	19 (11.3%)	0.224
Diabetes mellitus, n (%)	35 (10.6%)	14 (8.6%)	21 (12.5%)	0.255
Malignancy (previous or current), n (%)	27 (8.2%)	15 (9.3%)	12 (7.1%)	0.483
URTI: (n=309) Rhinitis, n (%) (n=300) Laryngitis/pharyngitis, n (%) (n=302) Sinusitis, n (%) (n=301) Otitis media, n (%) (n=302) Tonsilitis, n (%) (n=304)	276 (89.3%) 262 (87.3%) 109 (36.1%) 105 (34.9%) 30 (9.9%) 39 (12.8%)	128 (88.3%) 122 (87.1%) 50 (35.2%) 57 (40.1%) 19 (13.5%) 19 (13.2%)	148 (90.2%) 140 (87.5%) 59 (36.9%) 48 (30.2%) 11 (6.8%) 20 (12.5%)	0.576 0.926 0.764 0.071 0.054 0.857
LRTI: (n=309) Bronchitis, n (%) (n=300) Pneumonia, n (%) (n=306) Pleuritis, n (%) (n=302)	96 (31.1%) 89 (29.7%) 10 (3.3%) 5 (1.6%)	44 (30.6%) 40 (28.6%) 5 (3.5%) 3 (2.1%)	52 (31.5%) 49 (30.6%) 5 (3.1%) 2 (1.2%)	0.856 0.698 0.833 0.543
Subjective susceptibility to recurrent RTIs (n=302) History of >= 3 severe RTIs in the past (n=307) History of RTI lasting > 4 weeks (n=308) History of hospital treatment for infection (n=302)	78 (25.8%) 44 (14.3%) 59 (19.2%) 55 (18.2%)	39 (27.9%) 20 (14.1%) 30 (21.0%) 20 (16.5%)	39 (24.1%) 24 (14.5%) 29 (17.6%) 35 (21.7%)	0.454 0.909 0.449 0.090
Antibiotic treatment: (n=306)				
>6 courses	6 (2%)	3 (2.1%)	3 (1.8%)	0.858
4-6 courses	10 (3.3%)	5 (3.5%)	5 (3.0%)	
1-3 courses	123 (40.2%)	53 (37.6%)	70 (42.4%)	
non	167 (54.6%)	80 (56.7)	87 (52.7%)	
IgG, g/l, mean (SD) (normal range 7-16g/l) (n=320)	11.3 (2.9)	11.8 (2.74)	10.9 (2.99)	0.007
IgA, g/l, mean (SD) (normal range 0.7-4g/l) (n=312)	2.73 (1.24)	2.65 (1.15)	2.80 (1.33)	0.259
IgM, g/l, mean (SD) (normal range 0.4-2.3g/l)(n=310)	0.96 (0.54)	1.03 (0.61)	0.92 (0.47)	0.064
Hypogammaglobulinemia (IgG<7g/l), n (%) (n=320)	11 (3.4%)	5 (3.25%)	6 (3.61%)	0.857

For parameters, for which data from individual patients were missing, the n number for the individual item is given on the left. SD standard deviation, n number, BMI body mass index, csDMARD conventional synthetic disease modifying antirheumatic drug, bDMARD biological DMARD, NSAID non-steroidal anti-inflammatory drug, URTI upper respiratory tract infection, LRTI lower respiratory tract infection.

### Statistics

To summarize the baseline demographic and disease characteristics of the study cohort descriptive statistics were used (mean, standard deviation, percentages). To compare axSpA and PsA patients, Student’s t-tests were employed for normally distributed continuous variables, whereas Welch’s t-test was used for variables with unequal variance. To compare categorical variables between the groups the chi-square test was employed. Univariate and multivariate logistic regression were used to determine factors associated with LRTIs and antibiotic treatment. Multivariate models were created stepwise using backwards elimination after adjusting for demographic and disease factors. The statistical level of significance was set at p<0.05. All statistical calculations were performed using Jamovi version 2.0.0.0. (The jamovi project (2021). *jamovi*. (Version 2.2) [Computer Software]. Retrieved from https://www.jamovi.org ) (21).

## Results

### Patient cohort

Within this project a cohort of 330 spondyloarthritis patients was analysed, comprising 162 axSpA and 168 PsA patients. 55.5% of patients were male, 44.5% female. Mean age at time of analysis was 53.3 years and mean disease duration was 11.3 years. Epidemiological data are summarized in **Table 1**. 52.5% of patients within this cohort were HLA B27 positive. 22.2% of patients were active smokers, in the axSpA subgroup 29% and thus significantly more than in the PsA subgroup. Regarding clinical manifestations, 83.2% of patients had peripheral arthritis, 66.9% had axial involvement, 40.7% enthesitis and 25.5% dactylitis.

At the time of assessment, 80.5% of patients had a DMARD therapy. 34.5% of patients were treated with a csDMARD, 68.2% had a biologic therapy. The most commonly used biologics were TNF-alpha inhibitors (64.4%) and IL17 inhibitors (29.8%). 72/224 (32.1%) of patients on biologicals had a combination therapy with a csDMARD. 10.6% of patients conducted a monotherapy with NSAIDs. 8.8% of patients in this cohort were taking glucocorticoids (GC), with a mean daily dose of 6.05mg (min. 0.5mg, max 20mg). In total, patients had on average 2.1 previous DMARD therapies (min. 0, max. 12) and one previous biological therapy (min. 0, max. 8).

### Previous infection history

Out of 330 patients, 18.2% had a history of at least one hospital admission for treatment of infection. 19.2% of patients reported a respiratory tract infection of more than 4 weeks duration and 14.3% had a previous history of 3 or more severe respiratory infections. In total, 25.8% of patients reported feeling susceptible to recurrent airway infections. Subjective susceptibility to respiratory tract infections did not differ significantly between axSpA and PsA patients.

### Frequency of respiratory tract infections

**Figure 1:**
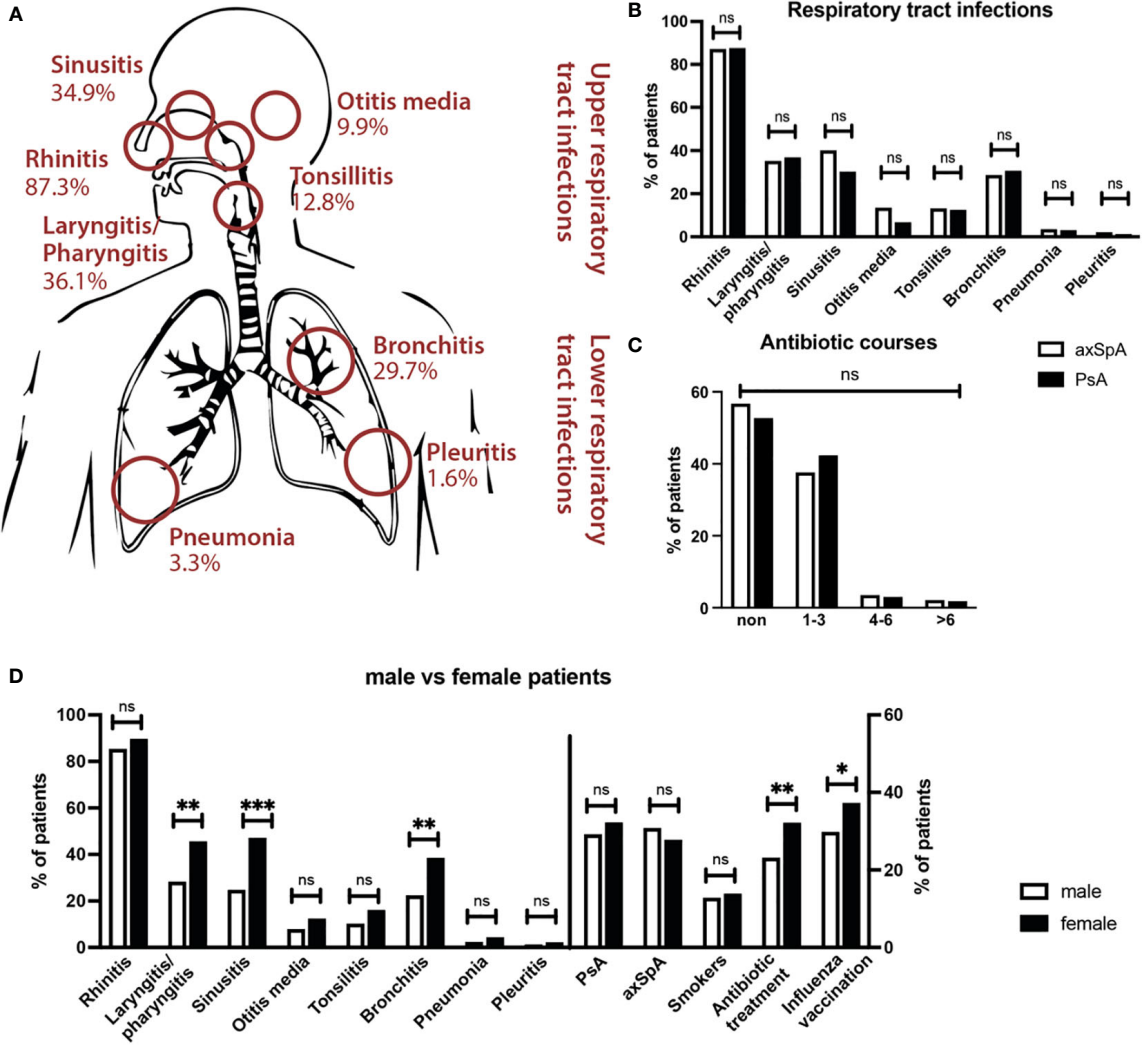
Frequency of respiratory tract infections in SpA patients. **(A)** Frequencies of upper and lower respiratory tract infections in total cohort. Rhinitis, laryngitis/pharyngitis and sinusitis were the most common infections. **(B)** Comparison of frequency of reported RTIs in axSpA (white bars) and PsA (black bars) shows no significant differences. **(C)** No significant differences in antibiotic therapy between axSpA (white) and PsA (black) patients. **(D)** Differences in infection frequency between male (white bars) and female patients (black bars). Female patients reported significantly more laryngitis/pharyngitis, sinusitis and bronchitis, and more often received antibiotic treatment *** p<0.001; ** p<0.01; * <0.05; ns not significant.

Respiratory tract infections reported for the timeframe of two years prior to the survey were quantified as following: 89.3% of patients reported a history of upper respiratory tract infections (URTI). The most commonly reported URTI was rhinitis with 87.3% of patients reporting at least one episode within the last two years. Pharyngitis/laryngitis occurred in 36.1% of patients and sinusitis in 34.9%. 12.8% of patients had a history of tonsillitis and 9.9% of otitis media. 31.1% of patients reported suffering from lower respiratory tract infections (LRTI). The most common LRTI was bronchitis, occurring in 29.7% of patients. Out of these, 18.0% of patients reported >3 episodes. Pneumonia was rare with an overall percentage of 3.3% of patients reporting at least one episode. Only one patient (0.3%) reported more than three episodes of pneumonia. At 1.6% pleuritis was the least common LRTI. Patient characteristics and exact frequency of distinct URTI/LRTI are listed in **Table 1** and shown in **Figure 1**. There were no statistically significant differences between axSpA and PsA, regarding both, overall URTI/LRTI numbers as well as any individual upper or lower respiratory tract infections.

Within the timeframe of two years 45.4% of patients had required antibiotics for respiratory tract infections, 88.5% of these reported 1-3 courses, 7.2% 3-6 courses and 4.3% more than 6 courses. There were no significant differences between axSpA and PsA patients regarding antibiotic therapy.

### Factors associated with infections and antibiotic therapy

Female patients reported a significantly higher frequency of LRTI (p=0.006). In particular, bronchitis (p=0.002) as well as sinusitis (p<0.001) and pharyngitis/laryngitis (p=0.002) were reported more commonly in female patients. Female patients also more often reported feeling susceptible to respiratory infections (p<0.001) and more commonly had a history of recurrent respiratory infections with >3 serious RTIs (p=0.026) as well as RTIs lasting >4 weeks (p=0.001). Furthermore, female patients had a significantly higher rate of antibiotic therapy compared to male patients (53.6% vs. 38.6%, p=0.009).

In this cohort, smoking was associated with LRTIs (p=0.013) and increased antibiotic treatment (p=0.007), but not with reported occurrence of URTIs. Specifically, smoking was associated with the occurrence of pneumonia (p=0.025) and pleuritis (p=0.030). Furthermore, smokers more commonly reported a previous history of multiple severe RTIs (p<0.001).

In general, patients suffering from LRTIs were significantly older (56.8 vs. 52.6 years, p=0.011), had more functional impairment in everyday life through their underlying SpA (p<0.001) and a reduced health-related quality of life (p<0.001) compared to patients without LRTIs.

A binomial logistic regression model was calculated to determine predictors independently associated with the occurrence of lower respiratory tract infections. A stepwise backwards elimination approach was used after adjusting for demographic and disease-related factors (age, sex, BMI, smoking status, diagnosis, disease duration, csDMARD therapy, biological therapy) as well as comorbidities (chronic lung disease, chronic kidney disease, previous or current malignant disease, diabetes mellitus). Chronic lung disease (p=0.006), glucocorticoid therapy (p=0.012), previous history of severe airway infections (p=0.013), number of previous biological therapies (p=0.017), as well as absence of HLA B27 (p=0.025) were independently associated with reported occurrence of LRTIs (**Table 2**).

**Table 2:** Multivariate logistic regression analysis of factors associated with LRTIs.

predictors	OR	95% CI	*B*	p
Chronic lung disease	17.44	2.31 – 131.74	2.86	0.006
GC therapy	9.24	1.65 – 51.88	2.22	0.012
History of ≥ 3 serious RTIs in past	6.82	1.49 – 31.20	1.92	0.013
N° of previous biological therapies	1.72	1.10 – 2.68	0.54	0.017
HLA B27	0.29	0.10 – 0.85	-1.24	0.025

Multivariable logistic regression adjusted for age, sex, BMI, smoking status, diagnosis, disease duration, csDMARD therapy, biological therapy) as well as comorbidities (chronic lung disease, chronic kidney disease, previous or current malignant disease, diabetes mellitus). GC glucocorticoid, N° number.

None of the individual disease manifestations axial involvement, peripheral arthritis, enthesitis or dactylitis were associated with an increase in reported occurrence of respiratory tract infections for the overall patient cohort. Patients with a disease duration of >10 years reported a subjectively increased susceptibility to RTIs (p=0.014), although in this cohort neither a short (<5 years) nor a long disease duration (>10 years) showed a clear association with an increased frequency of respiratory tract infections (data not shown).

Patients with chronic lung disease more often reported a history of multiple severe or prolonged respiratory tract infections in the past (p<0.001, p=0.012) as well as an increased subjective susceptibility to RTIs (p<0.001). Chronic lung disease was associated with an increased frequency of LRTIs in general (p<0.001), sinusitis (p=0.003), pharyngitis/laryngitis (p=0.010), tonsillitis (p=0.006) and bronchitis (p<0.001). Furthermore, patients with chronic lung disease received more antibiotic courses (p=0.002). Chronic kidney disease, malignancies as well as diabetes mellitus were not associated with more reported respiratory infections in this cohort.

A stepwise backwards elimination binomial logistic regression model showed antibiotic therapy to be independently associated with age (p=0.030), smoking (p=0.008), biologic therapy (p=0.004) and poor sleep quality (assessed by RIS score, p<0.001) after controlling for demographic and disease-associated factors (sex, BMI, diagnosis, disease duration, HLA-B27 status, functional capacity), as well as comorbidities (chronic lung disease, chronic kidney disease, previous or current malignant disease, diabetes mellitus). GC and DMARD therapy as well as history of previous severe infections did not show any significant association with antibiotic therapy (**Table 3**).

**Table 3:** Multivariate logistic regression analysis of factors associated with antibiotic therapy.

	OR	95% CI	*B*	p
Smoking	3.40	1.38 – 8.36	1.22	0.008
Biological therapy	3.38	1.47 – 7.79	1.22	0.004
RIS score	1.13	1.05 – 1.21	0.12	<0.001
Age	0.96	0.93 – 0.99	-0.04	0.030
GC therapy	1.39	0.40 – 4.82	0.33	0.603
csDMARD therapy	1.54	0.70 – 3.40	0.43	0.284
History of ≥ 3 serious RTIs in past	1.52	0.42 – 5.55	0.42	0.524
History of hospital admission for infection	1.64	0.62 – 4.37	0.50	0.319

Multivariable logistic regression adjusted for diagnosis, disease duration, sex, BMI, HLA-B27 status, FFbH score, chronic lung disease, chronic kidney disease, previous or current malignant disease, diabetes mellitus. GC glucocorticoid, RIS Regensburg Insomnia Scale, FFbH Funktionsfragebogen Hannover.

As the regression model showed a negative association of antibiotic therapy with age, we stratified antibiotic intake by age group (**Table 4**). Indeed, the age group of 20-29-year-olds had the highest rate of antibiotic therapy (66.67% within the time frame of 2 years), although LRTIs were actually less frequently reported than in other age groups. However, the subgroup of 20-29-year-olds had the highest rate of bDMARD therapy compared to other age groups (95%).

**Table 4:** Stratification of antibiotic therapy and infections by age group.

Age group	N°	% w AB	% w LRTIs	% w URTIs	% w bDMARD
**20-29**	20	66.67%	22.22%	94.44%	95.00%
**30-39**	44	37.84%	10.81%	97.22%	68.18%
**40-49**	52	52.00%	32.65%	90.00%	69.23%
**50-59**	94	42.53%	32.18%	87.50%	70.21%
**60-69**	88	49.40%	38.17%	84.71%	62.50%
**70-79**	25	29.17%	36.00%	91.30%	64.00%
**80-89**	7	28.60%	28.57%	83.33%	42.86%

N° number of patients, w with, AB antibiotic therapy

### Immunomodulatory therapy

Glucocorticoid therapy was associated with LRTIs (p=0.039), specifically with reported occurrence of bronchitis (p=0.044) as well as frequency of pneumonia (p=0.008). Despite a low dose, patients taking GC were two times more likely to have reported LRTIs.

Current methotrexate therapy was not associated with reported respiratory tract infections or increased frequency of antibiotic therapy in this cohort. Regarding biological therapies, patients with a history of hospital admission for infections more often received anti-IL17 treatment (p=0.014). Anti-IL17 therapy was consequently associated with increased LRTIs (p=0.011), occurrence of pneumonia (p=0.002) as well as antibiotic therapy (p=0.008). Anti-TNF treatment was not associated with increased infections or antibiotic treatment in this cohort. However, patients on anti-TNF treatment were significantly younger than patients without anti-TNF therapy (p<0.001).

The number of previous therapies was associated with previous history of infections, but also with frequency of respiratory tract infections in the two years prior to the survey: Patients with ≥ 3 previous DMARD therapies had a higher frequency of otitis (p=0.010) and pharyngitis/laryngitis (p=0.007) as well as an increased number of antibiotic courses (p=0.002). Patients with ≥ 3 previous DMARD therapies were, however, also more likely to receive glucocorticoid treatment (p=0.014).

Similarly, ≥ 3 previous biological therapies were associated with a history of inpatient treatment for infection (p=0.001), subjective susceptibility to RTIs (p=0.046) as well as URTIs (p=0.027) and LRTIs (p=0.020). In particular, reported rhinitis (p=0.014), pharyngitis/laryngitis (p<0.001), bronchitis (p=0.007) and flu-like infections (p=0.026) occurred more frequently in this group.

### Immunoglobulin concentrations

Serum immunoglobulin concentrations were available for 320 patients and are listed in **Table 1**. Mean serum IgM for the overall cohort was 0.98g/l (normal range 0.4-2.3g/l), mean IgG 11.3g/l (normal range 7-16g/l), and mean IgA 2.73g/l (normal range 0.7-4g/l). The most commonly observed change was a reduction of serum IgM concentrations, occurring in 9.7% of patients (minimum 0.22g/l). IgM deficiency was associated with a reported history of RTI > 4 weeks duration (p=0.038). Patients with IgM reduction were significantly older (59.8 vs. 52.85 years, p=0.009). IgM reduction was associated with age (p=0.004) and smoking (p=0.043). Mean IgG concentrations were significantly lower in PsA than in axSpA patients (10.88g/l vs. 11.75g/l, p=0.007). Hypogammaglobulinemia (<7g/l) was generally rare and was observed in only 3.44% of patients, while severe hypogammaglobulinemia (IgG <4g/l) did not occur in any patient. No patient in this cohort received immunoglobulin replacement therapy. Hypogammaglobulinemia was associated with a previous history of severe RTIs as well as history of RTI >4 weeks duration in the past (both p<0.001), occurrence of pneumonia (p=0.006) as well as an increased number of antibiotic courses (p=0.012). IgA deficiency was rare, occurring in 1% of patients. A simultaneous reduction of ≥ 2 immunoglobulin classes was noted in only 3 patients (1%).

### Infection prevention measures

55.4% of patients in this cohort reported receiving yearly influenza vaccinations (female 62.1%, male 49.7%, p=0.029). Patients with influenza vaccination were significantly older (57.9 vs. 49.21 years, p<0.001) and had a longer disease duration (12.5 vs 9.82 years, p=0.014). Patients on csDMARD therapy more often received influenza vaccination (p=0.004), whereas in this cohort biological therapies were not associated with higher vaccination rates. In the subgroup of patients >60 years of age, 70.1% of patients were vaccinated against influenza. Influenza vaccination status was associated with a reported history of severe respiratory tract infections as well as an increased subjective susceptibility to respiratory infections (p=0.003, respectively). Patients with yearly influenza vaccination had higher rates of reported URTIs (p=0.035), LRTIs (p=0.009), rhinitis (p=0.025), and bronchitis (p<0.001).

51.5% of patients in this cohort received vitamin D supplementation. Patients on vitamin D supplementation were significantly older (56.0 vs. 50.5 years, p<0.001), more likely female (p<0.001) and more likely to suffer from PsA than axSpA (p=0.012). Vitamin D supplementation was associated with glucocorticoid therapy (p=0.002) as well as with an increased reported occurrence of URTI and LRTI (p=0.039, respectively p=0.013) and antibiotic therapy (p=0.023).

## Discussion

In this study we assessed the frequency of respiratory infections and determined associated factors in a real-world monocentric cohort of 330 spondyloarthritis patients. While respiratory tract infections in general were frequently reported, severe respiratory infections were rare in this cohort. Occurrence of LRTIs was associated with chronic lung disease, glucocorticoid treatment, number of previous biological therapies and previous history of severe infections in this cohort, whereas HLA B27 positivity was associated with a decreased rate of RTIs. This is in line with data published by Bon San Koo et al. and Moura et al., who described age, glucocorticoid therapy, previous history of serious infections and a higher number of previous doctors’ visits as risk factors for (severe) infections in TNF-treated SpA patients (22, 23). Similar findings for rheumatoid arthritis were published by Strangfeld et al. as well as Singh et al., showing GC therapy, age, functional capacity, serious infections in the previous 12 months and comorbidities such as COPD/chronic lung disease and chronic kidney disease to be associated with an increased infection risk (6, 7). These overarching risk factors for infections thus seem independent of the individual rheumatologic diagnosis. However, specific therapies may increase the individual risk in predisposed patient groups, such as those with comorbidities.

In general, the overall infection risk in spondyloarthritis patients (axSpA, PsA) is assumed to be lower than in rheumatoid arthritis (17, 24, 25), which may be attributed to younger age, differences in treatment, but also infection risk conferred by the disease itself (24). Only few studies exist, which compare infection risk in axSpA and PsA. Quartuccio et al. described similar risks for serious infections for axSpA and PsA (1). Similarly, in our cohort we did not observe significant overall differences between axSpA and PsA patients regarding respiratory infections. The burden of respiratory tract infections was not generally elevated in our cohort of SpA patients compared to the general population (18).

We observed an independent association of lower respiratory tract infections with GC therapy (OR 9.24) despite a low GC dose of on average 6.05mg prednisone/day. Immunosuppressive therapies constitute an important risk factor regarding frequency of infection, and especially glucocorticoids are well-known to be associated with an increased infection risk (26–28). Previous studies showed that the association of GC therapy with infections is dose-dependent, increases with duration of GC therapy and exists independently of the rheumatologic diagnosis (26, 29, 30). More recently, studies have confirmed a significant albeit small risk of serious infection even at low doses of 5 mg prednisone equivalent or less per day (29, 31), which is consistent with our results. In contrast, csDMARD therapy did not show a significant influence on infection risk in this study. For RA patients, it had been demonstrated that nonbiologic DMARDs including methotrexate do not significantly increase the risk of infection (27, 32). However, a high number of previous DMARD therapies does seem to contribute to infection risk in RA (7). On the other hand, biological therapies and especially TNF antagonists may increase the risk of serious infections, especially in the first months of treatment (16). Quartuccio et al. described a 2-fold increase in the risk for hospitalisation due to infection after the start of biological therapy in a cohort consisting of RA, SpA, PsA and psoriasis patients (1). However, most infectious episodes arising under biological therapy were reported to be minor (8, 33). There are only few published studies on respiratory tract infections under biologicals other than anti-TNF, such as anti-IL17, and most data are derived from clinical trials. Wan et al. described a potentially increased risk of respiratory infections under IL17 inhibition in a meta-estimate calculated for phase 3 trials in psoriasis patients, however, concluded that more detailed studies were needed (34). URTI were among the most common side effects of anti-IL17 phase III trials across all indications (35, 36). Furthermore, rhinitis and nasopharyngitis not necessarily of infectious origin, were commonly reported across the phase 3 studies (37). However, severe infections were rare in the clinical trials (36). In our cohort, anti-IL17 therapy was, however, not only associated with URTI but also with occurrence of LRTIs and antibiotic intake. However, patients on anti-IL17 therapy also more often had a previous history of severe infection. Therefore, a channelling bias might be at least partially responsible for this observation, as patients with recurrent infections on previous therapies may rather be started on anti-IL17 than anti-TNF medication in clinical practice. Further studies will be needed to elucidate the effects of anti-IL17 treatment on infection risk in real life.

In addition to immediate effects on the immune system long-term immunosuppressive therapy may lead to secondary antibody deficiency predisposing to recurrent infections. Especially glucocorticoids, B-cell-directed therapies as well as csDMARDs, such as methotrexate and sulfasalazine have been shown to cause hypogammaglobulinemia over time (15, 38, 39). There are only few data available for non-B-cell-directed biological therapies, but hypogammaglobulinemia seems to be rare. Furthermore, there are only few studies published on immunoglobulin levels in SpA or PsA, most of which predate the current therapeutic approaches and recommendations (40–42). Reports on hypogammaglobulinemia in PsA/SpA exist mostly in the form of case reports (43, 44). Antibody deficiency was rare in this cohort with hypogammaglobulinemia occurring in only 3% of patients and generally being mild despite an average disease duration of 11.3 years. None of the patients in this cohort had significant hypogammaglobulinemia of <4g/l. This is in contrast to other rheumatic conditions such as rheumatoid arthritis and small vessel vasculitides, in which secondary hypogammaglobulinemia occurs more frequently, which may in part be attributed to different therapeutic regimens, but may also be associated with immunological processes inherent to the underlying rheumatic condition (45).

Within this cohort, we observed a higher rate of reported LRTIs, but also certain URTIs in female patients, who also more often reported a history of recurrent or prolonged infections in the past. This phenomenon has also been observed by Moura et al. and Germano et al., who similarly described a higher infection risk for female patients (2, 23). Whereas the other authors speculated that this was explained due to more urinary tract infections in women, our data indicate that also RTIs are increased. Therapies may constitute a mediating factor regarding gender differences in infection risk. Female patients in our cohort slightly more often had a csDMARD or glucocorticoid therapy (37.7% vs 32.4%, respectively 10.3% vs 7.8%) and were slightly less frequently treated with a biological therapy (64.6% vs 70.9%), though all not statistically significant. Furthermore, the fact that immunosuppressive therapies in inflammatory arthritides are not adjusted to body weight may explain part of the gender differences regarding infection risk as the on average lower body weight of female patients may lead to relative overtreatment.

Furthermore, chronic lung disease was associated with a higher frequency of reported respiratory infections. This association has been previously shown for RA patients as well as the general population (18, 46).

In contrast, HLA B27 positivity was an independent protective factor for LRTIs in this study, indicating that HLA B27 might confer a protective effect against LRTIs. Evidence from the literature suggests that HLA B27 confers a degree of protection for viral infections, such as HIV and hepatitis C as well as possibly to influenza (47–49). Thus, a role of HLA B27 in anti-viral immune response may account for the observed inverse relationship with reported airway infections seen in this cohort. Further studies will be needed to elucidate the effect of HLA B27 on respiratory infections.

Interestingly, whereas current biological therapy was not associated with an increased occurrence of LRTIs, biological therapy did show an independent association with reported antibiotic treatment (OR 3.38) in a multivariate regression model. Conversely, GC treatment was independently associated with occurrence of LRTIs, however, this did not translate into an association with antibiotic use. This observation suggests that prescribing doctors, who are mostly general practitioners in Germany, evaluate the risk of infection under individual immunomodulatory/immunosuppressive therapies differently, potentially attributing a higher risk to biological therapies. Thus, patient and healthcare professional education and awareness regarding risk of infection and antibiotic use under immunosuppressive therapy are crucial. The topic of infection prevention and control has gained novel interest during the COVID19 pandemic. In this real-world patient cohort, preferentially older patients had been vaccinated against influenza (70% of patients aged ≥60 years), thus influenza vaccination was not in general associated with a reduced number of reported infections in this cohort. Possibly those with more frequent infections in the past or a predisposition towards infections were more likely to get vaccinated. In addition, the fact that only a small percentage of all RTIs is due to influenza may explain why influenza vaccination does not show a relevant protective effect for general RTIs. Out of the total cohort only 55% of patients were vaccinated against influenza. This rather low frequency of influenza vaccination is in spite our department’s policy to strongly recommend vaccinations according to the updated EULAR recommendation, stating that vaccination should be strongly considered for all rheumatic patients (50). Our data also indicate that the humoral immune system is rarely compromised in axSpA/PsA patients under immunomodulatory treatment and thus, a good protection can be expected after vaccination in this patient group, rendering vaccination an easy and effective preventive measure.

Furthermore, 22.2% of patients from the overall cohort and 29% from the axSpA subgroup were active smokers, which replicates published results from the international ASAS-COMOSPA study with also 29% of smoking prevalence in axSpA patients (51). Smoking has been associated with higher disease activity and poorer quality of life in SpA (52) in addition to being a risk factor for respiratory infections (10).

Preventive measures such as vaccinations against influenza, pneumococcal disease and SARS-CoV2 as well as smoking cessation should thus be strongly encouraged, especially in patients with other risk factors for infections such as older age, previous serious infections or glucocorticoid therapy. Furthermore, tapering and, if possible, cessation of glucocorticoid therapy should be enforced to reduce modifiable risk factors for infections. Furthermore, patient education of vulnerable patient groups regarding hand hygiene, cough etiquette, contact avoidance, wearing medical masks at crowded locations or social distancing can contribute to infection prevention.

Weaknesses of this study include the comparatively small patient cohort owing to the fact that this was a monocentric study, with not enough observed events in case of rare events such as pneumonias to reach sufficient statistical power. A further limitation of this study is the questionnaire-based approach, which may lead to recall bias. However, this issue was addressed by additional medical chart analysis of patients’ notes and medical reports, where at least more serious infections are noted. However, the very fact that this was a single-center study also means that detailed clinical information on all patients was available and data density was high with only few missing data, and lack of inter-center variability. Furthermore, it should be pointed out that this study largely took place during the COVID19 pandemic. We did not detect any statistically significant differences in infection frequencies over time during the study period, however, it cannot be ruled out that COVID prevention measures such as face masks, social distancing, and improved hand hygiene had an influence on infection frequency.

In conclusion, in this retrospective analysis we determined the frequency of respiratory infections and associated factors in a real-world monocentric cohort of 330 spondyloarthritis patients. We identified multiple factors associated with increased risk of respiratory tract infections. Awareness of these risk factors will assist physicians and other health professionals to identify and monitor high-risk patients in the population who will benefit from additional preventive measures or adjustment of DMARD therapy.

## Data availability statement

The raw data supporting the conclusions of this article will be made available by the authors, without undue reservation.

## Ethics statement

The studies involving human participants were reviewed and approved by the Ethics committee of the University of Freiburg, Germany. The patients/participants provided their written informed consent to participate in this study.

## Author contributions

NV, RV, JT, AN participated in the design and supervision of the study and gave critical input. ER, CH, MD, NB, NF and NV carried out patient recruitment and consenting, provided clinical information and cared for the patients enrolled in this study. NF, ER, RL, AV and NV performed data analysis and interpretation. NF and NV wrote the manuscript. All authors contributed to the article and approved the submitted version.

## Funding

Parts of this study were financially supported by an unrestricted grant by Novartis Pharma GmbH, Germany. The funder was not involved in the study design, collection, analysis, interpretation of data, the writing of this article or the decision to submit it for publication.

## Acknowledgments

We would like to thank all patients who participated in this study.

## Conflict of interest

NV: Speaker honoraria: AbbVie, Novartis, UCB, Bristol-Myers-Squibb, Pfizer; Advisory Boards: AbbVie, Novartis, UCB; Research grants: Bristol-Myers-Squibb, Novartis, Pfizer. JT: Speaker honoraria: GSK, BMS, Astra-Zeneca, Abbvie, UCB, Lilly; Advisory Boards: Novartis, GSK, Astra-Zeneca, Lilly. Grant/research support from: BMS, Novartis. RV: Speaker fees: AbbVie, Amgen, BMS, Boehringer-Ingelheim, GSK, Janssen-Cilag, Hexal, Novartis, Pfizer, Roche; Advisory boards: AbbVie, Amgen, Boehringer-Ingelheim, BMS, GSK, Janssen-Cilag, Hexal, Neutrolis, Novartis, Sanofi, Takeda; Unrestricted research grants: Amgen, BMS, Novartis, Pfizer. MD and NF received travel grants from Pfizer, Janssen, Sobi.

The remaining authors declare that the research was conducted in the absence of any commercial or financial relationships that could be construed as a potential conflict of interest.

## Publisher’s note

All claims expressed in this article are solely those of the authors and do not necessarily represent those of their affiliated organizations, or those of the publisher, the editors and the reviewers. Any product that may be evaluated in this article, or claim that may be made by its manufacturer, is not guaranteed or endorsed by the publisher.
